# Identification of *Respiratory Burst Oxidase Homolog* (*Rboh*) Family Genes From *Pyropia yezoensis* and Their Correlation With Archeospore Release

**DOI:** 10.3389/fpls.2022.929299

**Published:** 2022-07-12

**Authors:** Tian-Yu Gui, Da-Hai Gao, Hong-Chang Ding, Xing-Hong Yan

**Affiliations:** ^1^Key Laboratory of Exploration and Utilization of Aquatic Genetic Resources, Ministry of Education, Shanghai Ocean University, Shanghai, China; ^2^Shanghai Aquaculture Engineering Technology Research Center, Shanghai Ocean University, Shanghai, China

**Keywords:** *Pyropia yezoensis*, archeospore, RBOH gene, DPI treatment, evolutionary analysis

## Abstract

Reactive oxygen species (ROS) play important regulatory roles in plant growth and development, as well as in cell differentiation and stress responses. Respiratory burst oxidase homolog (RBOH) is the key enzyme in ROS production. So far, the *Rboh* family genes in *Pyropia yezoensis* have not been comprehensively characterized, and whether their function was involved in the formation of archeospores is still unknown. In this study, a total of 11 *PyRboh* genes were identified from the *P. yezoensis* genome by homology mining. Through phylogenetic analysis, it is suggested that the *PyRboh* genes were evolutionarily conserved among the lineages of red algae, but a few genes exhibited a species-specific manner. The treatment of *P. yezoensis* blades with NADPH oxidase inhibitor diphenylene iodonium (DPI) could significantly inhibit the formation of archeospores, suggesting that RBOH may be involved in the formation of archeospores. According to *PyRboh* gene expression analysis using the *P. yezoensis* strains with obvious differences in releasing archeospores, it is showed that the expression trends of most genes were consistent, with no significant difference between strains, whereas the expression pattern of the two *P. yezoensis*-specific genes (*PyRbohJ* and *PyRbohK*) was positively correlated with the amount of archeospores. Furthermore, as treatment of blades with allantoin resulted in a significant increase in the release of archeospores, the expression levels of *PyRbohJ* and *PyRbohK* were also consistently upregulated, further confirming the relationship between the two genes and archeospore formation. These findings provide insights into the molecular mechanism of *P. yezoensis* archeospore formation.

## Introduction

Reactive oxygen species (ROS), including superoxide (${\text{O}}_{2}^{-}$), singlet oxygen (^1^O_2_), and hydrogen peroxide (H_2_O_2_), are important signaling molecules in living creatures (Kaur and Pati, 2016). In plants, ROS have a regulatory role in growth and development, as well as the processes of cell differentiation and stress responses *via* the ROS signaling network (Kiyono et al., 2021). The level of ROS in cells is critically determined by the synergy between ROS production pathways and antioxidant mechanisms (Herb et al., 2021). Intertidal red algae respond to a variety of intense environmental challenges during their growth and maturation, relying mostly on complex antioxidant processes to maintain the homeostasis of ROS (Shim et al., 2022). The current studies on ROS in red algae are mainly focused on the activity and the corresponding gene expression of antioxidant enzymes during stress, such as superoxide dismutase (SOD), catalase (CAT), ascorbate peroxidase (APX), and glutathione reductase (GR) (Choi et al., 2018).

Respiratory burst oxidase homolog (RBOH, also known as NADPH oxidase) is a key enzyme of the ROS production pathway and a key node for the ROS signaling network (Marino et al., 2012; Wang et al., 2020b). As a class of transmembrane protein, RBOH mediates the transfer of electrons from intracellular NADPH to extracellular O_2_, resulting in the production of ${\text{O}}_{2}^{-}$, which is then catalyzed to H_2_O_2_ by SOD (Suzuki et al., 2011). In terms of protein structure, the C-terminal region of the RBOH contains the transmembrane domains and the functional oxidase domains, and the N-terminal region is composed of calcium-binding EF-hand motifs and phosphorylation domains (Chang et al., 2020). Therefore, RBOH is also considered to be associated with the intracellular calcium signaling pathway. In angiosperms, the *Rboh* genes belong to a multi-copy gene family, and a total of 10, 9, and 13 *Rboh* genes were identified from model species of *Arabidopsis thaliana, Oryza sativa*, and *Zea mays*, respectively (Groom et al., 1996; Torres and Dangl, 2005; Wong et al., 2007; Lin et al., 2009; Kimura et al., 2020). Several studies have indicated that *Rboh* family genes are always distinct in spatiotemporal gene expression and their enzymatic activity, which means that the function of these paralogous genes is differentiated (Li et al., 2015). In red algae, a total of 2, 10, and 7 *Rboh* genes have been identified in *Cyanidioschyzon merolae, Pyropia haitanensis*, and *Porphyra umbilicalis*, respectively (Cao et al., 2020), whereas the *Rboh* gene family in *P*. *yezoensis* has not been comprehensively characterized.

As an important cultivated red alga, *P*. *yezoensis* is mainly distributed along the coasts of China, Japan, and the Korean Peninsula, the life history of which is composed of two heterogeneous generations of gametophytic blade and sporophytic conchocelis (Wu et al., 2017). There are sexual and asexual reproduction modes in *P*. *yezoensis*, with the latter producing gametophytic clones directly into gametophytic blades *via* the asexual spores called archeospores (Kong et al., 2014). In terms of field application, the archeospores of *P*. *yezoensis* are of great significance for assisting the development of seedlings, prolonging the harvest period, and increasing yield (Zhang et al., 2014). Studies have proved that high light, high temperature, low salt, and exogenous additives (e.g., allantoin) can promote the formation and release of archeospores (Song et al., 2020), but less is known about the underlying molecular mechanism.

Through comparative transcriptome analysis, our laboratory found that the expression of some *Rboh* genes in *P*. *yezoensis* was significantly upregulated during the process of archeospore formation. Therefore, whether this family is involved in the formation and release of archeospores is worthy of investigation. In this study, we identified *Rboh* family genes from *P*. *yezoensis* and analyzed their evolutionary patterns. In addition, the effect of RBOH enzyme inhibitor [diphenylene iodonium (DPI)] treatment on the releasing archeospores was observed, and *Rboh* genes associated with archeospore formation were screened using the gene expression analysis. This study demonstrated a potential role of *Rboh* genes in the process of archeospore formation, which will provide a reference for deciphering the molecular mechanism of asexual reproduction in *P*. *yezoensis*.

## Materials and Methods

### Algal Strains, Culture Condition, and Pharmaceutical Treatment

The *P*. *yezoensis* strains of *Py26W* and *Py-LS* with scarce archeospores and the strains of *Py26W'* and *Py-332* with abundant archeospores were used in this study. The blades were cultured in sterile seawater with MES medium (renewed every 3 days) under 40 μmol photons/(m^2^·s) light density in a photoperiod of 10 L:14 D conditions at the temperature of 19 ± 1°C. All of the above strains were preserved in the laboratory in the form of conchocelis, and the preservation methods were described by Chen et al. (2016).

Diphenylene iodonium is a specific inhibitor of the RBOH enzyme, and the optimal concentration of DPI in cultured blades of the 20-day-aged *Py-332* strain has been determined to be 0.05 μM based on preliminary experiments. In the treatment group, the blades were treated with 0.05 μM DPI for 3 days and then recovered in the normal medium without DPI for 7 days, whereas in the control group, the blades were cultured in the medium without DPI during the whole experimental period. The number of archeospores released from the blades was analyzed in the following methods.

Allantoin, 2,5-dioxo-4-imidazolidinyl urea, is an intermediate metabolite in plant purine catabolism. Previous studies have shown that allantoin treatment can significantly promote the formation and release of archeospores (Mizuta et al., 2003). In this study, the optimum concentration of allantoin for *Py-332* strain is 5 mM based on preliminary experiment and the 22-day-aged blades were used for allantoin treatment. The number of archeospores released from the blades was analyzed by following the methods. The above exogenous treatment experiments were all set up in three parallel groups.

### Quantification of Archeospores

The blades used to quantify the archeospores were cultured in a plastic cup containing a 200-ml culture medium. The plastic cup was replaced once a day, and the blades were moved into a new plastic cup for subsequently culturing. The released archeospores attached to the cup wall and the gap tube were brushed and transferred to a Petri dish, which were then counted under a microscope. The quantification includes 5 blades and 3 parallel groups, which was normalized as a single blade per day.

### Genes Identification and Bioinformatic Analysis

The reference genome sequences of *P*. *yezoensis, P*. *haitanensis, P*. *umbilicalis, Gracilariopsis chorda, C. merolae, Chondrus crispus, Porphyridium purpureum, Chlamydomonas reinhardtii, and O. sativa* were acquired from the NCBI database (https://www.ncbi.nlm.nih.gov/). The sequences of the *Arabidopsis* RBOH were obtained from the TAIR database (https://www.arabidopsis.org/), and the BLAST tool was used for homology alignment analysis. The candidate sequences of RBOH screened from the above genomes were submitted to the HMMER website (https://www.ebi.ac.uk/Tools/hmmer/) and the SMART website (http://smart.embl-heidelberg.de/) to verify the conserved domains of NAD and FAD in protein sequences. The molecular weight, instability index, and PI of putative RBOHs were analyzed using the online software of Protparam (http://web.expasy.org/protparam/). In addition, protein subcellular localization prediction was carried out using the online software of WoLFPSORT (https://wolfpsort.hgc.jp/), CELLO (http://cello.life.nctu.edu.tw/), Plant-mPLoc (http://www.csbio.sjtu.edu.cn/bioinf/plant-multi/), and Yloc (https://www.yloc.org/en/).

### Sequence Alignment and Phylogenetic Analysis

The Clustal method (Larkin et al., 2007) was used to align RBOH proteins from the above species. The phylogenetic trees were constructed using neighboring joining (NJ), maximum likelihood (ML), and Bayesian inference (BI) methods, respectively. NJ tree was constructed using the MEGA7.0 software (Kumar et al., 2016), with 1,000 bootstraps. ML tree was constructed using IQtree (Nguyen et al., 2015), with the model of “LG+G” and 500 bootstraps. BI tree was constructed suing the MrBayes software (Ronquist and Huelsenbeck, 2003), with the model of “HKY,” and MCMC parameters were set as follows: generations to 2,000,000, sampling frequency to 100, number of runs to 2, and number of chains to 4.

### Conserved Motifs, Domains, and Chromosomal Localization Analysis

The conserved motifs of Rboh protein were analyzed using the MEME software (Bailey et al., 2009), and the parameters were set as follows: the number of motifs was 12, and the optimal sequence width of amino acid residues was from 6 to 50. The conserved NAD and FAD domains of Rboh protein sequences were aligned using the MEGA X-ClustalW software, and the results were visualized using the TBtools software (Chen et al., 2020). Chromosomal localization was performed using the gene location visualize module of the TBtools software, and the location of *Rboh* genes was visualized based on annotation information of *P*. *yezoensis*. Non-synonymous (Ka) and synonymous (Ks) substitution rates were calculated using the simple Ka/Ks calculator module of the TBtools software.

### Gene Expression Analysis

Total RNA was extracted from blades using TRIzol reagent (Thermo Fisher Scientific) and reverse transcribed into cDNA with PrimeScript™ RT Reagent Kit and gDNA Eraser Kit (TaKaRa). Then, the Bio-Rad CFX96 PCR instrument and SYBR I fluorescent dye (TaKaRa) were used for real-time quantitative PCR. Primers were designed using the Primer Premier 5 software (Lalitha, 2000) based on the CDS sequence of the identified *PyRboh* genes (Supplementary Table S1), and *Py-UBC* was used as an internal reference gene (Kong et al., 2015). The PCR reaction system and program settings were referred to the study by Li et al. (2019), and the 2^−ΔΔ*CT*^method was used to calculate the relative expression of genes. For each reaction, 3 biological and 3 technical replicates were included.

### Statistical Analysis

The experimental data were processed using the Excel 2010 software, and the values were expressed as mean ± SD. The independent sample *t*-test of SPSS version 18.0 was used to test the differences between groups, and *P* < 0.05 and *P* < 0.01 were considered significant difference and extremely significant difference, respectively.

## Results

### Identification of *Rboh* Genes From *Pyropia yezoensis*

BLAST homologous searching initially obtained 11 *Rboh* genes from the genome of *P*. *yezoensis*. HMMER and SMART analysis verified that all of these corresponding Rboh proteins have conserved characteristic domains of NAD and FAD, confirming that the *Rboh* gene family of *P*. *yezoensis* contains 11 members. According to the nomenclature rule of the plant *Rboh* gene family, the members of the *Rboh* gene family in *P*. *yezoensis* were named *PyRbohA*-*K*. As a common feature of red algal Rboh proteins, PyRboh proteins lack an EF-hand motif which serves as a calcium-binding site (Shim et al., 2022).

**Figure 1:**
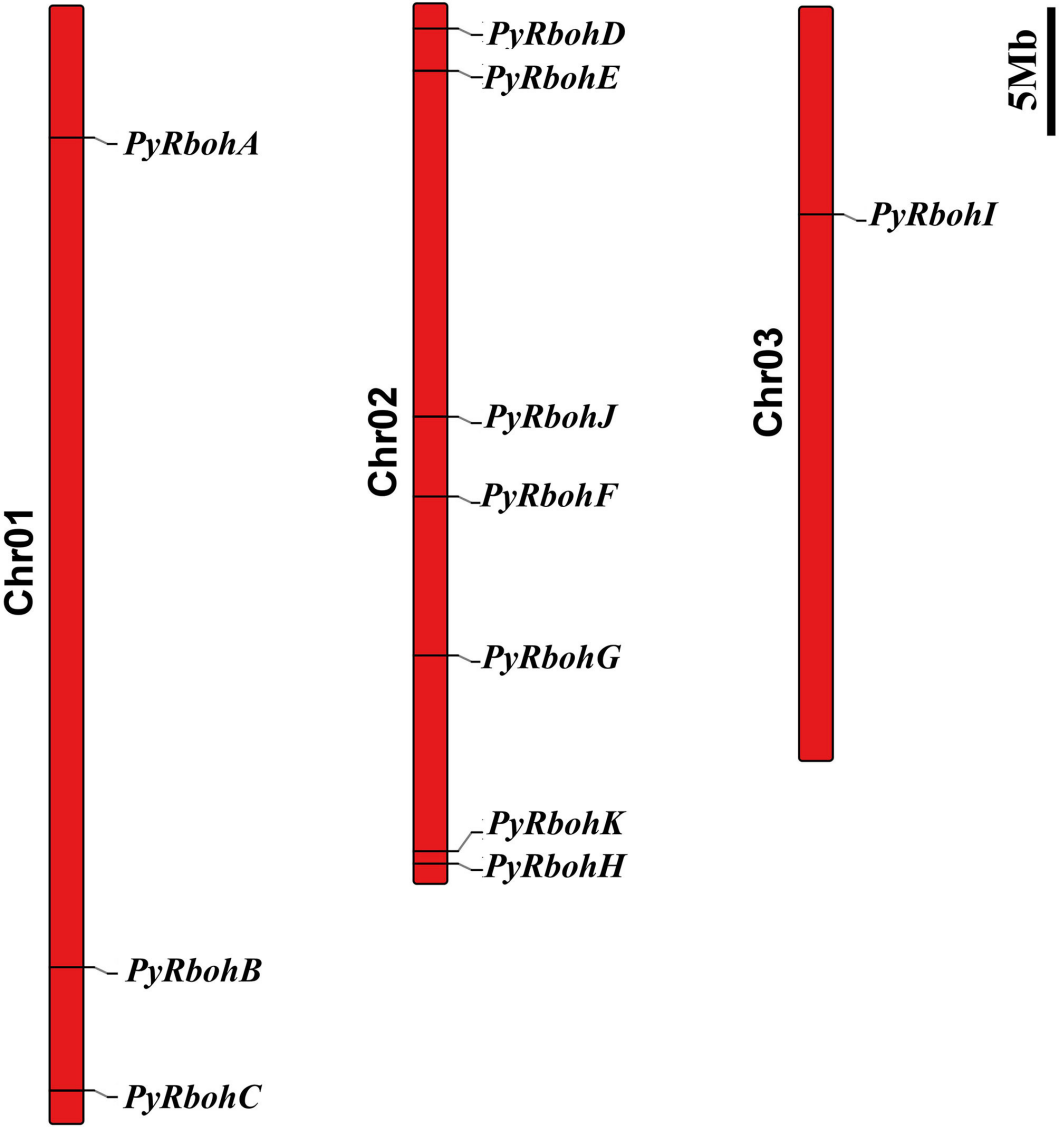
The chromosomal locations of the *PyRboh* genes. The long red bars represent the chromosomes of *P. yezoensis*. The chromosome numbers are labeled on the left of the bars, and the black fonts represent the *PyRboh* genes.

Based on physicochemical properties' analysis of PyRboh protein, it is showed that molecular weight was from 58.41 to 144.30 kDa, the PI value was from 6.51 to 10.36, the fat coefficient was from 77.58 to 101.97, and the instability coefficient was between 33.29 and 48.32 (Supplementary Table S2). Furthermore, the subcellular localization prediction revealed that similar to that of other species, most PyRboh proteins were localized in the plasma membrane (Supplementary Table S3). Chromosomal localization analysis indicated that the *PyRboh* genes were scattered on three chromosomes of *P*. *yezoensis* (Figure 1), with *PyRbohA*-*C* on Chr01; *PyRbohD*-*H, PyRbohJ*, and *PyRbohK* on Chr02, and *PyRbohI* on Chr03.

### Phylogenetic and Evolutionary Analyses of the Rboh Proteins

**Figure 2:**
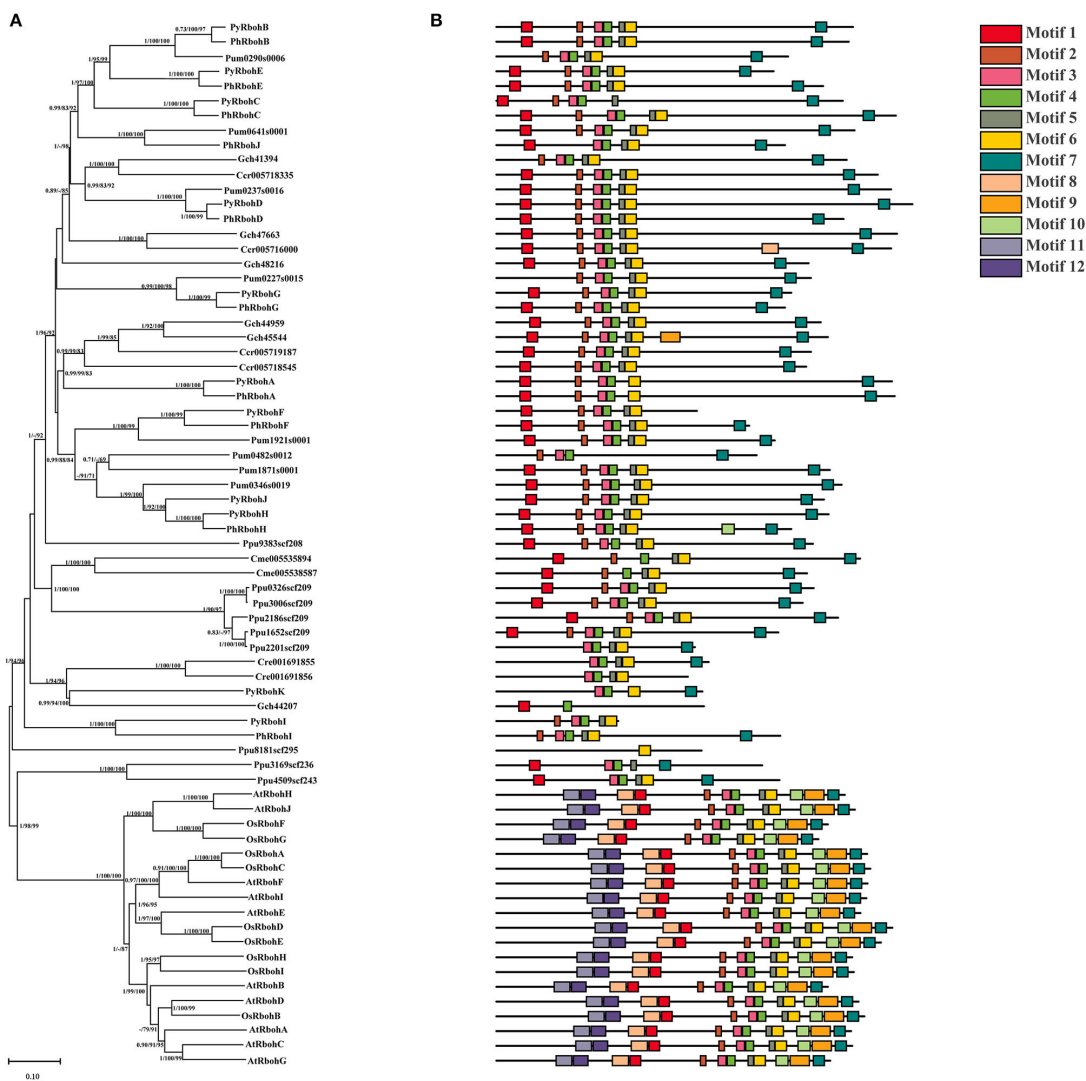
Phylogenetic tree **(A)** and conserved motifs **(B)** of Rboh proteins from representative species of red algae, green algae, and land plants. Species' name abbreviations are used as prefixes: Ph, *Pyropia haitanensis*; Py, *Pyropia yezoensis*; At, *Arabidopsis thaliana*; Os, *Oryza sativa*; Cre, *Chlamydomonas reinhardtii*; Ccr, *Chondrus crispus*; Cme, *Cyanidioschyzon merolae*; Gch, *Gracilariopsis chorda*; Pum, *Porphyra umbilicalis*; Ppu, *Porphyridium purpureum*.

A total of 50 Rboh proteins were identified from 7 red algae species (*P*. *yezoensis, P*. *umbilicus, P*. *haitanensis, C. merolae, G. chorda, C. crispus*, and *P. purpureum*) and two proteins were identified from green alga species (*C. reinhardtii*). Based on phylogenetic analysis, it is showed that 10 AtRboh proteins (*A. thaliana*) and 9 OsRboh proteins (*O. sativa*) clustered as a monophyletic clade at the base of the tree and most Rboh proteins from red algae and *C. reinhardtii* were grouped together (Figure 2A). In Rhodophyta, the evolution of the Rboh proteins is generally conserved, as indicated by the consistent phylogenetic relationship with species evolutionary relationship. Besides, according to motif analysis, the positions of some motifs matched with the conserved regions of FAD and NAD, with motifs 4–7 constituting the FAD domain and motifs 10–12 constituting the NAD domain. In addition, the motif types, numbers, and positions of the Rboh proteins in the same phylogenetic clades were similar (Figure 2B).

**Figure 3:**
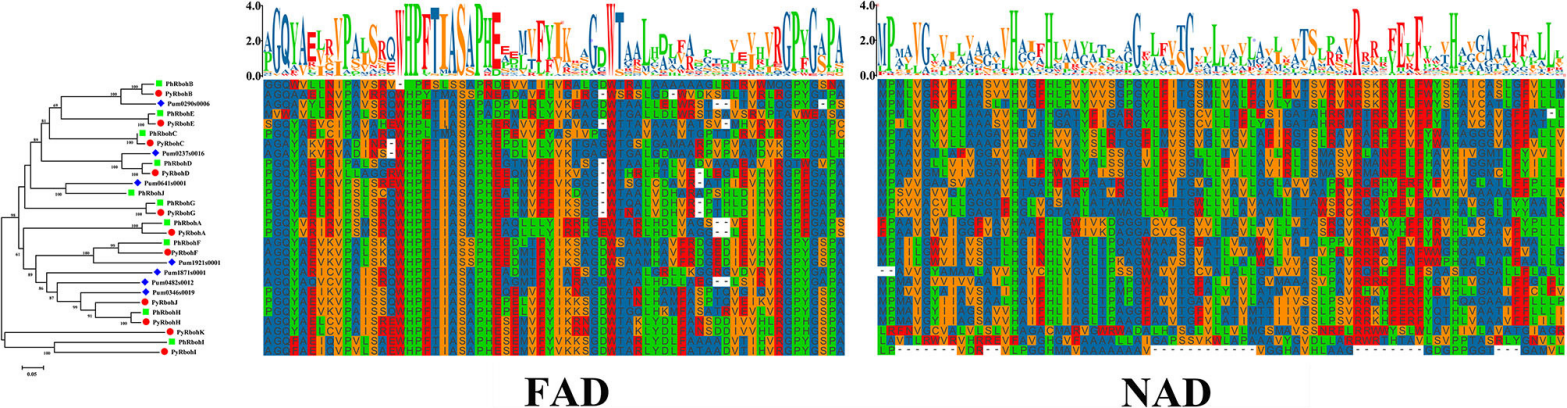
Phylogenetic analysis and sequence alignment of FAD and NAD conserved regions of Rboh proteins in *P. yezoensis, Pyropia haitanensis*, and *Porphyra umbilicalis*. The colored shapes next to the tree branches represent different species. FAD, FAD binding domain; NAD, NAD binding domain.

To elucidate the evolutionary relationship of Rboh proteins from *P*. *yezoensis, P*. *haitanensis*, and *P*. *umbilicalis*, phylogenetic relationships based on 28 conserved FAD and NAD sequences of Rboh were further analyzed (Figure 3). It is showed that the 9 Rboh proteins from *P*. *yezoensis* (PyRbohA-PyRbohI) exhibited a 1:1 orthologous relationship with that from *P*. *haitanensis* (PhRbohA-PhRbohI). In contrast, *P*. *yezoensis-*specific PyRbohJ and PyRbohK and *P*. *haitanensis-*specific PhRbohJ have also been identified. In addition, 3 of the 7 Rboh proteins from *P*. *umbilicalis* were on the same orthologous clades as *P*. *yezoensis* and *P*. *haitanensis*, respectively. Based on the selection pressure analysis of 9 orthologous pairs between *P*. *yezoensis* and *P*. *haitanensis*, it is showed that Ka was much smaller than Ks and all Ka/Ks were smaller than 0.2 (Table 1), indicating that these genes were evolved under strong purifying selection.

**Table 1:** Ka, Ks, and Ka/Ks values among orthologous genes in *Pyropia yezoensis* and *Pyropia haitanensis*.

**Seq 1**	**Seq 2**	**Ks**	**Ka**	**Ka/Ks**
*PyRbohA*	*PhRbohA*	1.0877	0.1106	0.1016
*PyRbohB*	*PhRbohB*	0.7962	0.0343	0.0431
*PyRbohC*	*PhRbohC*	0.9786	0.1034	0.1057
*PyRbohD*	*PhRbohD*	0.9475	0.0617	0.0651
*PyRbohE*	*PhRbohE*	1.0412	0.1007	0.0967
*PyRbohF*	*PhRbohF*	1.4409	0.1371	0.0952
*PyRbohG*	*PhRbohG*	0.0671	0.9430	0.0712
*PyRbohH*	*PhRbohH*	0.9788	0.0791	0.0808
*PyRbohI*	*PhRbohI*	0.5218	0.0492	0.0943

### Effect of DPI Treatment on Archeospore Release

**Figure 4:**
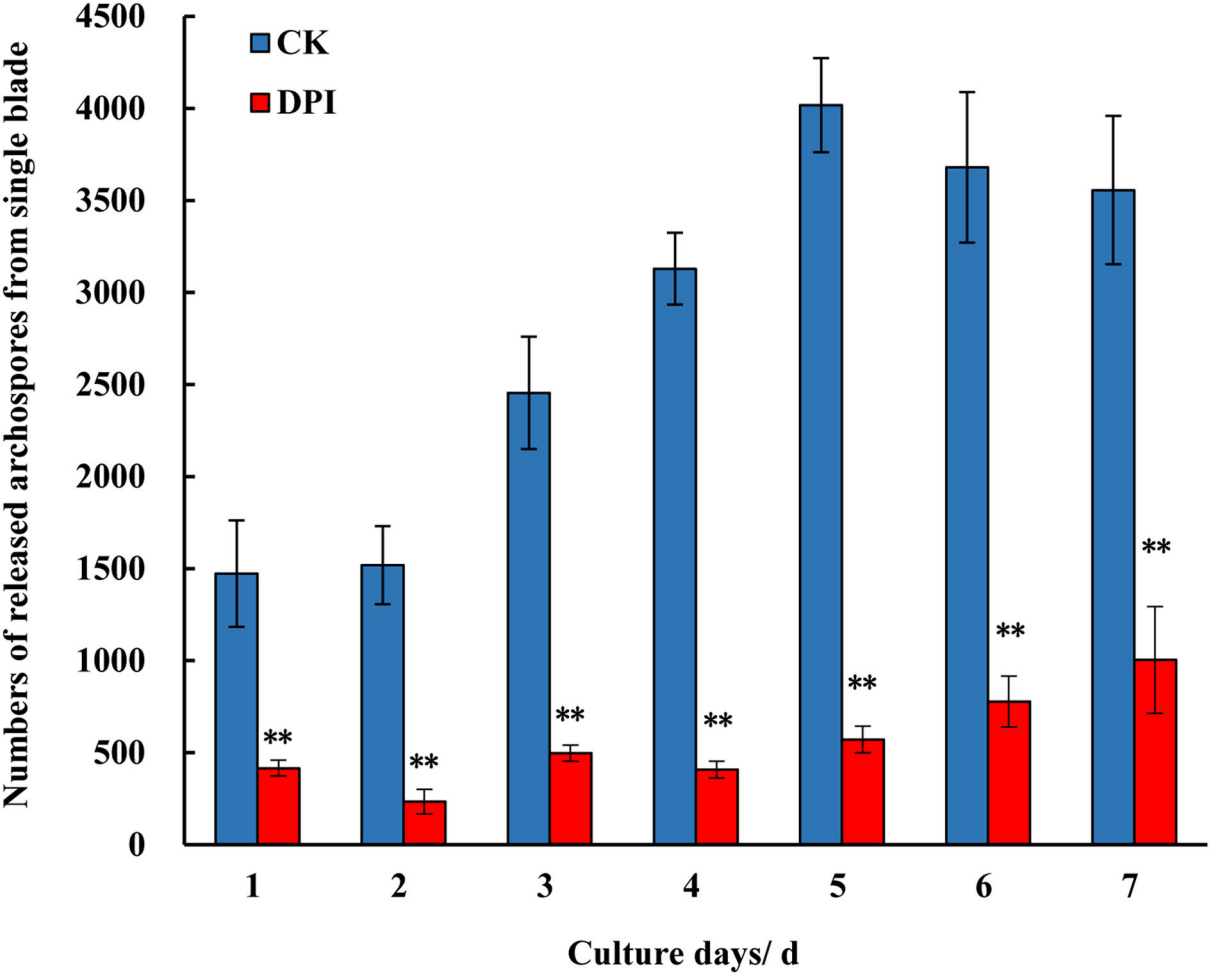
Numbers of the archeospores released from the blades of *P. yezoensis Py-332* strain after being cultured in normal medium (CK) and DPI (0.05 μM) medium (DPI) for 3 days and then cultured in normal medium for another 7 days. **Extremely significant difference (*P* < 0.01).

As DPI is widely used as a specific inhibitor of the RBOH enzyme, DPI treatment could test the physiological effect when the activity of RBOH is blocked. In the treatment group of *Py-332* strain, the formation and release of archeospores were suppressed distinctively during 7 days of recovery after treatment, with the daily amount of archeospores from a single blade being significantly lower than that of the control group at each day point (Figure 4). Meanwhile, in the control group, a large number of archeospores were released from blades, with the daily release amount of a single blade reaching about 4,000 at day 5.

### Identification of *PyRboh* Genes Correlated With Archeospore Release

**Figure 5:**
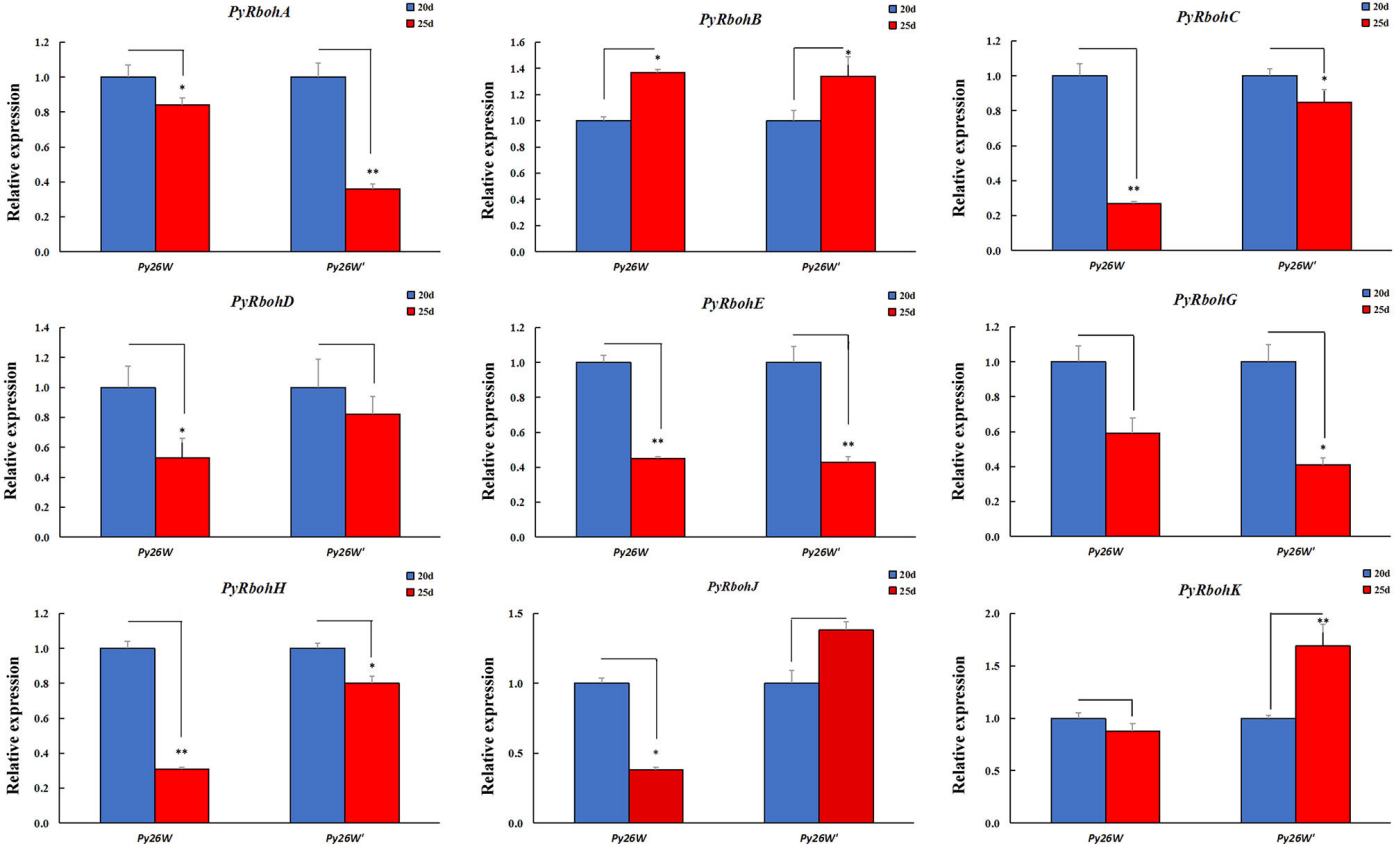
Expression trends of *PyRboh* genes for blades of *P*. *yezoensis* at the age of 20 and 25 days in *Py26W* and *Py26W'* strains. *Significant difference (*P* < 0.05); **Extremely significant difference (*P* < 0.01).

To identify the *PyRboh* genes associated with the process of archeospore release, the expression trends for *PyRboh* genes were compared by investigating the strains with different abilities in archeospore release. The strains of *Py26W* and *Py26W'* were isolated *via* intraspecific hybridization in our laboratory, with the former releasing scarce archeospores and the latter releasing abundant archeospores. In addition, it found that most genes were expressed differently between strains (Supplementary Figure S2). For the expression trend analysis at the age of 20 and 25 days, it is indicated that the expressions of *PyRbohC*-*E, PyRbohG*, and *PyRbohH* showed a consistent downregulation trend in the two strains, and the expression of *PyRbohB* showed a consistent upregulation trend (Figure 5).

**Figure 6:**
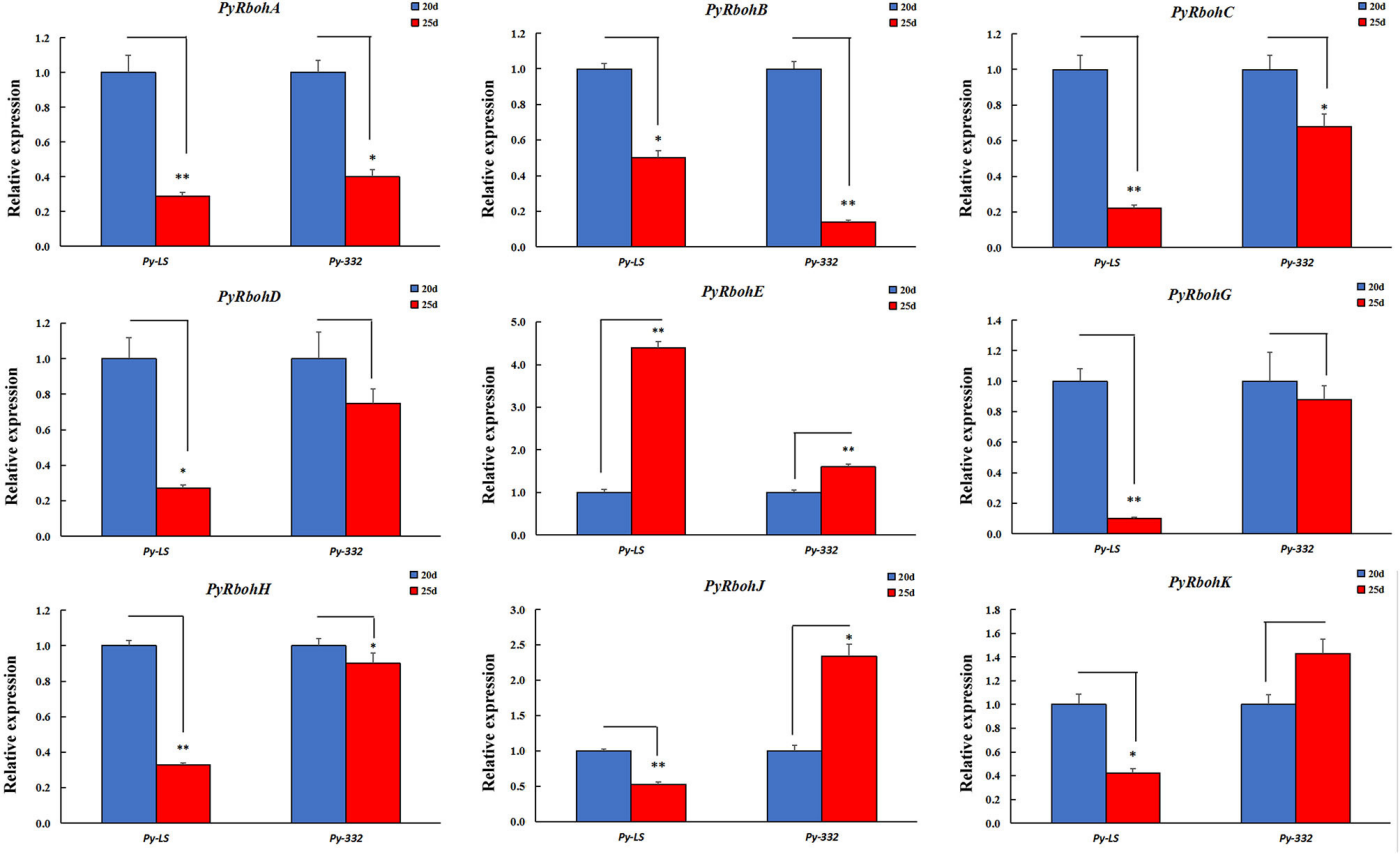
Expression trends of *PyRboh* genes for blades of *P*. *yezoensis* at the age of 20 and 25 days in *Py-LS* and *Py-332* strains. *Significant difference (*P* < 0.05); **Extremely significant difference (*P* < 0.01).

Another two strains of *Py-LS* and *Py-332* were purified wild-type strains by our laboratory, with the former basically releasing few archeospores and the latter releasing abundant archeospores. It also found that most genes were expressed differently between strains (Supplementary Figure S3). For the expression trend analysis at the age of 20 and 25 days, it is indicated that the expression of *PyRbohA*-*D, PyRbohG*, and *PyRbohH* in the two strains showed a consistent downregulation trend, and *PyRbohE* showed a consistent upregulation trend (Figure 6). Interestingly, the expression of *PyRbohJ* and *PyRbohK, two P*. *yezoensis*-specific genes, was both upregulated in the archeospores-abundant strains and downregulated in the archeospores-scarce strains (Figures 5, 6). As the expression trends of these two genes were correlated with the feature of archeospore formation, it is suggested that their functions may be related to the formation and release of archeospores.

### Gene Expression of *PyRbohJ* and *PyRbohK* Under Allantoin Treatment

**Figure 7:**
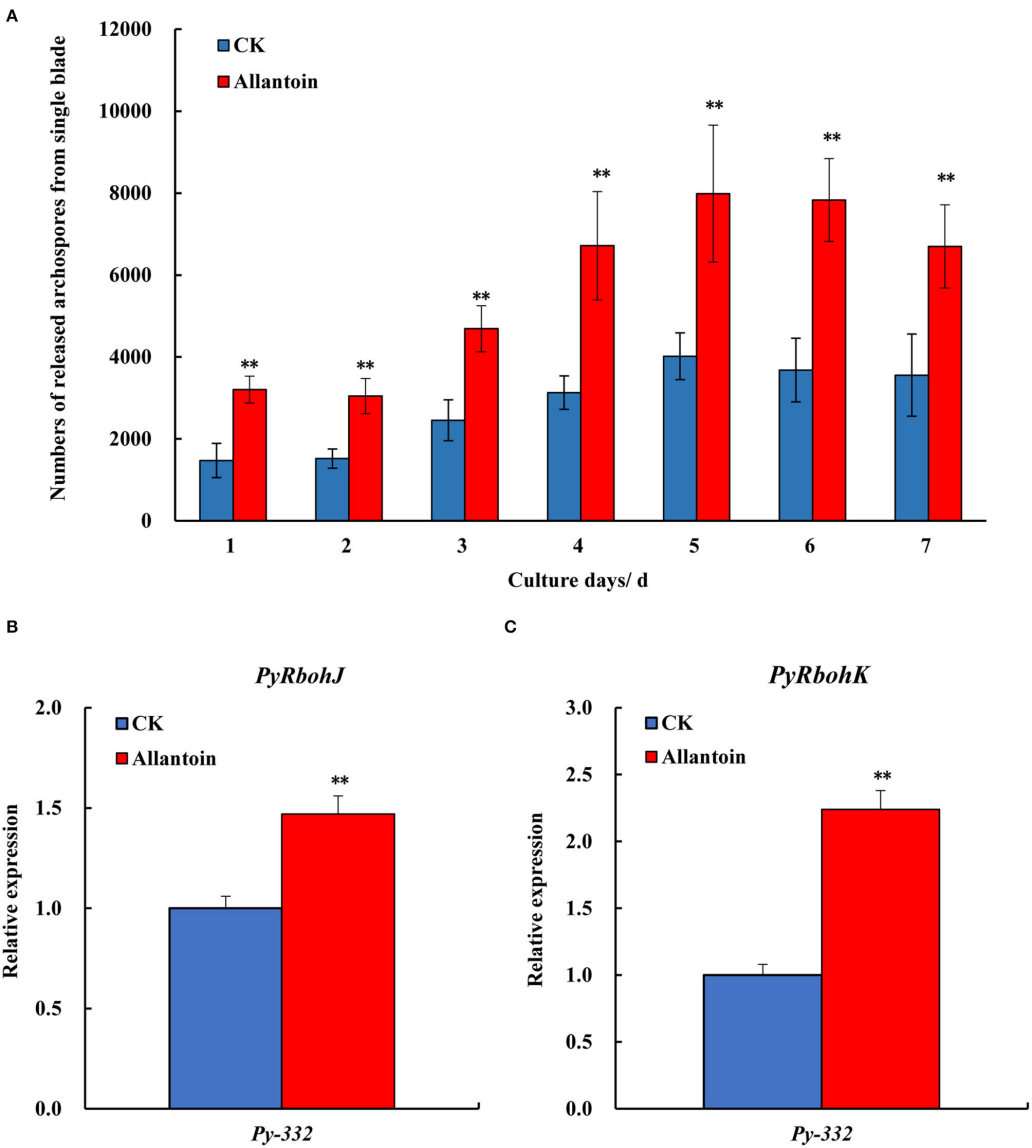
Numbers of the released archeospores from the 22-day-aged blades after being cultured in normal medium (CK) and allantoin (5 mM) medium (Allantoin) for 7 days **(A)** and the relative expression of *PyRbohJ* **(B)** and *PyRbohK* **(C)** of the blades in *Py-332* strain of *P. yezoensis*. **Extremely significant difference (*P* < 0.01).

To verify the correlation between the expression trend of *PyRbohJ* and *PyRbohK* and the formation of archeospores, the *Py-332* strain was treated with 5 mM allantoin. During the 7 days of treatment, the number of archeospores released from the blades was significantly higher in the experimental group than in the control group (*P* < 0.01) (Figure 7A), confirming that allantoin treatment could accelerate the formation and release of archeospores. In addition, the relative expression levels of *PyRbohJ* (Figure 7B) and *PyRbohK* (Figure 7C) genes were also significantly higher in the experimental group than in the control group after 7 days of treatment (*P* < 0.01).

## Discussion

In plants, RBOH is the key enzyme in the ROS production pathway and a key node in the ROS signaling network (Wong et al., 2004). The *Rboh* genes belong to a multi-copy gene family and have been comprehensively identified and analyzed in model species such as *A. thaliana* and *O. sativa*. However, few studies on the *Rboh* genes were carried out in red algae. In this study, a total of 11, 10, and 8 *Rboh* genes were identified from the genomes of *P*. *yezoensis, P*. *haitanensis*, and *P*. *umbilicalis*, respectively. Although the number of *Rboh* genes in these bladed Bangiales species is similar to the abovementioned model plants, the phylogenetic analysis showed that the *Rboh* genes derived from red algae are distantly related to land plants (Figure 2A). As the differentiation time of red algae and land plants is long, the *Rboh* genes could be evolved independently. In addition, the number of *Rboh* genes identified from other species of the Rhodophyta was less than that of the Bangiales, which were 2, 4, and 6 *Rboh* genes from *C. merolae, C. crispus*, and *G. chorda*, respectively. We speculate that this number variation may be correlated with the genome size of species in the Rhodophyta. In contrast, related studies in model plants have shown that the *Rboh* gene family plays important regulatory roles in stress responses (Huang et al., 2021b), so the number of *Rboh* genes in Rhodophyta may be related to adapt to external environmental stress. Bladed Bangiales are distributed in the intertidal zone, subjected to various environmental conditions such as high temperature, high light, and high salt (Huang et al., 2021a), so more *Rboh* genes may be required to deal with the harsh environment. The genome-level comparative analysis found that large-scale gene family expansion and contraction events were prevalent in the genomes of Rhodophyta species, indicating that they formed specific genetic components under different environmental selection pressures to adapt to their respective ecological niches (Wang et al., 2020a).

As a representative lineage of Rhodophyta, issues such as the origin and evolution of bladed Bangiales and its stress tolerance mechanism have attracted much attention. The comprehensive identification and analysis of gene families provide a reference for it. This study further elucidates the evolutionary relationship of bladed Bangiales Rboh by constructing the phylogenetic tree of *P*. *yezoensis, P*. *haitanensis*, and *P*. *umbilicalis*. According to the phylogenetic relationships and species evolutionary relationships, the 9 Rboh proteins of *P*. *yezoensis* and *P*. *haitanensis* showed a 1:1 orthologous relationship (Figure 3), with strong purifying selection (Table 1), suggesting that the functions of these genes are relatively conserved. In plants, the *Rboh* genes are mainly involved in growth and development, cell differentiation, and stress responses (Liu et al., 2020). It is speculated that the conserved genes of bladed Bangiales may have common roles in adapting to the complex intertidal environment and regulating their own growth and development. In addition, *PyRbohJ* and *PyRbohK* are *P*. *yezoensis*-specific genes, and *PhRbohJ* is a *P*. *haitanensis*-specific gene, which may be involved in species-specific functions. For example, *P*. *yezoensis* can form and release archeospores in the blade period, whereas *P*. *haitanensis* does not possess this characteristic.

Diphenylene iodonium specifically inhibits the activity of the RBOH enzyme by reacting with the heme prosthetic group to form a stable adduct (Osaki et al., 2011), which has been widely used in plants (Huang et al., 2014; Liu et al., 2019; Navathe et al., 2019). Compared with the treatment methods in plants, the concentration and treatment time in this study were different. The DPI concentration of the culture medium used for treating *P. yezoensis* blades was 0.05 μM, and the treatment time was 3 days. However, higher concentration or longer treatment time was lethal to the cells of the blade, indicating that the function of RBOH is important to the basic physiology of *P. yezoensis*. It is also speculated that this phenomenon was related to the culturing feature of *P. yezoensis*, as the blades were completely immersed in a culture medium, infiltrating DPI into the cells more easily. In addition, when the blades were treated with DPI and then recovered for 5–7 days, the number of archeospores became increasing, suggesting that the inhibitory effect of DPI was reduced eventually (Figure 4).

Through the gene expression pattern analysis of compared strains with the release of abundant archeospores and scarce archeospores, we found that the expression patterns of *P. yezoensis*-specific *PyRbohJ* and *PyRbohK* genes were strongly correlated with the release patterns of archeospores (Figures 5, 6). Furthermore, it is showed that allantoin treatment resulted in promoting the release of archeospores, and the expression levels of two *P. yezoensis*-specific genes were also significantly upregulated simultaneously, confirming their correlation to the formation and release of archeospores (Figure 7). Studies have shown that exogenous addition of H_2_O_2_ can promote the release of archeospores, indicating that oxidative stress could induce vegetative cells to develop into archeospores (Takahashi and Mikami, 2017). In plants, H_2_O_2_, produced by the RBOH enzyme, is an important signaling molecule, which can regulate protein activity by interacting with -SH of cysteine, thereby initiating the cell response process (Zhang et al., 2022). Therefore, we deduced that the H_2_O_2_ produced by the specific *PyRbohJ* and *PyRbohK* might act as a signaling molecule, which induces the vegetative cell to develop into archeospores *via* ROS signaling networks and downstream regulation.

## Conclusion

A total of 11 genes in the *Rboh* family of *P. yezoensis* have been identified in this study, and *P. yezoensis*-specific *PyRbohJ* and *PyRbohK* are related to the formation and release of archeospores, which could provide insights for revealing the molecular mechanism of the formation and release of archeospores in *P. yezoensis*.

## Data Availability Statement

The original contributions presented in the study are included in the article/Supplementary Materials, further inquiries can be directed to the corresponding author.

## Author Contributions

D-HG and X-HY designed the study. T-YG conducted the experiments. T-YG and D-HG analyzed the data. T-YG, D-HG, and H-CD wrote the manuscript, which was revised by D-HG and X-HY. All authors read and approved the final version of the manuscript.

## Funding

This study was supported by the National Key Research and Development Program of China (2018YFD0900606), the Startup Foundation for Young Teachers of Shanghai Ocean University (2019), and the Open Program of Key Laboratory of Cultivation and High-Value Utilization of Marine Organisms in Fujian Province (2020fjscq01).

## Conflict of Interest

The authors declare that the research was conducted in the absence of any commercial or financial relationships that could be construed as a potential conflict of interest.

## Publisher's Note

All claims expressed in this article are solely those of the authors and do not necessarily represent those of their affiliated organizations, or those of the publisher, the editors and the reviewers. Any product that may be evaluated in this article, or claim that may be made by its manufacturer, is not guaranteed or endorsed by the publisher.

## Supplementary Material

Supplementary Table 1 PCR quantitative primer sequences for the *PyRboh* genes in *P. yezoensis*.

Supplementary Table 2 Physicochemical properties of PyRboh proteins in *P. yezoensis*.

Supplementary Table 3 Predicted subcellular localization of PyRboh proteins in *P. yezoensis*.

Supplementary Figure S1 Expression profiles of *PyRboh* genes in conchocelis and different developmental stages of blades in *P. yezoensis*.

Supplementary Figure S2 Expression profiles of *PyRboh* genes between *Py26W* and *Py26W'* strains at the age of 20 and 25 days, respectively. ^*^Significant difference (*P* < 0.05); ^**^Highly significant difference (*P* < 0.01).

Supplementary Figure S3 Expression profiles of *PyRboh* genes between *Py-LS* and *Py-332* strains at the age of 20 and 25 days, respectively. ^*^Significant difference (*P* < 0.05); ^**^Highly significant difference (*P* < 0.01).

Supplementary File S1 The information of the Rboh proteins in this study.
