# Supplementary material for: Identification of Respiratory Burst Oxidase Homolog (Rboh) Family Genes From Pyropia yezoensis and Their Correlation With Archeospore Release
Source: Front Plant Sci. 2022 Jul 12;13:929299. doi: 10.3389/fpls.2022.929299 (PMC9322803; doi:10.3389/fpls.2022.929299)
Supplement: Supplementary Table 1 — PCR quantitative primer sequences for the PyRboh genes in P. yezoensis. [file Data_Sheet_2.DOCX]

**Supplementary Table 1|** PCR quantitative primer sequences for the *PyRboh* genes in *Pyropia yezoensis*.

| **Gene name** | **Forward primer sequence (5’-3’)** | **Reverse primer sequence (5’-3’)** |
| --- | --- | --- |
| *PyRbohA* | ACGCTAACGCCCCTACC | ACCGCCCTCAGCACCA |
| *PyRbohB* | AAGCATCCATCTTGGGGGTG | GGTTGACACTCGCAAACCAC |
| *PyRbohC* | CCGCACGAGTCGGAGAT | CGCATTGGCAAACAGGTC |
| *PyRbohD* | CGCCACGACGACTACC | CGAGGAGCACCACCAG |
| *PyRbohE* | CCTTCCTGCTCGTCCTCT | CTTGTCAAACGGCACCAC |
| *PyRbohG* | CGCTGCTCAACCTCAACTC | ACGCACGCAACGACCT |
| *PyRbohH* | TGCGATGCGGATGGTG | GGCTCAGCGACGGGATA |
| *PyRbohJ* | GGGCTTTGGCTTCACT | CAGCAGGACGGACAGG |
| *PyRbohK* | TCACGCCCCTCCAGTC | CCAATCATCGCCACCTC |
| *Py-UBC* | TTTCCAAGGTGCTCCTCTCCATC | GGTCTCTTCATAGCGACTGCGGT |

**Supplementary Table 2|** Physicochemical properties of PyRboh proteins in *Pyropia yezoensis*.

| **Protein name** | **Molecular weight**  **(KDa)** | **pI** | **Instability index** | **Aliphatic index** | **Grand average of hydropathicity** |
| --- | --- | --- | --- | --- | --- |
| PyRbohA | 136.65 | 7.46 | 44.33 | 82.49 | 0.087 |
| PyRbohB | 103.13 | 9.22 | 43.37 | 92.39 | 0.047 |
| PyRbohC | 144.30 | 9.06 | 48.32 | 77.58 | 0.040 |
| PyRbohD | 119.76 | 7.11 | 45.48 | 89.98 | 0.096 |
| PyRbohE | 87.79 | 8.71 | 38.11 | 94.17 | 0.211 |
| PyRbohF | 95.03 | 6.51 | 35.92 | 91.35 | 0.105 |
| PyRbohG | 58.41 | 10.36 | 33.29 | 101.85 | 0.541 |
| PyRbohH | 87.20 | 10.30 | 42.12 | 100.61 | 0.279 |
| PyRbohI | 69.25 | 9.59 | 45.75 | 101.25 | 0.613 |
| PyRbohJ | 93.37 | 9.09 | 35.32 | 90.62 | 0.252 |
| PyRbohK | 61.04 | 8.97 | 34.68 | 101.97 | 0.438 |

**Supplementary Table 3|** Subcellular localization of PyRboh proteins in *Pyropia yezoensis*.

| **Protein name** | **WoLF PSORT** | **CELLO** | **Plant-mPLoc** | **Yloc** |
| --- | --- | --- | --- | --- |
| PyRbohA | Nucelar | Plasma membrane | Plasma membrane | Nucelar |
| PyRbohB | Plasma membrane | Plasma membrane | Plasma membrane | Chloroplast |
| PyRbohC | Plasma membrane | Nucelar | Plasma membrane | Plasma membrane |
| PyRbohD | Plasma membrane | Plasma membrane | Plasma membrane | Plasma membrane |
| PyRbohE | Plasma membrane | Plasma membrane | Plasma membrane | Chloroplast |
| PyRbohF | Plasma membrane | Nucelar | Plasma membrane | Chloroplast |
| PyRbohG | Plasma membrane | Plasma membrane | Plasma membrane | Plasma membrane |
| PyRbohH | Plasma membrane | Plasma membrane | Plasma membrane | Plasma membrane |
| PyRbohI | Plasma membrane | Nucelar | Plasma membrane | Plasma membrane |
| PyRbohJ | Plasma membrane | Plasma membrane | Chloroplast | Chloroplast |
| PyRbohK | Plasma membrane | Chloroplast | Chloroplast | Chloroplast |
