# Supplementary figures and images for: Identification of Respiratory Burst Oxidase Homolog (Rboh) Family Genes From Pyropia yezoensis and Their Correlation With Archeospore Release
Source: Front Plant Sci. 2022 Jul 12;13:929299. doi: 10.3389/fpls.2022.929299 (PMC9322803; doi:10.3389/fpls.2022.929299)

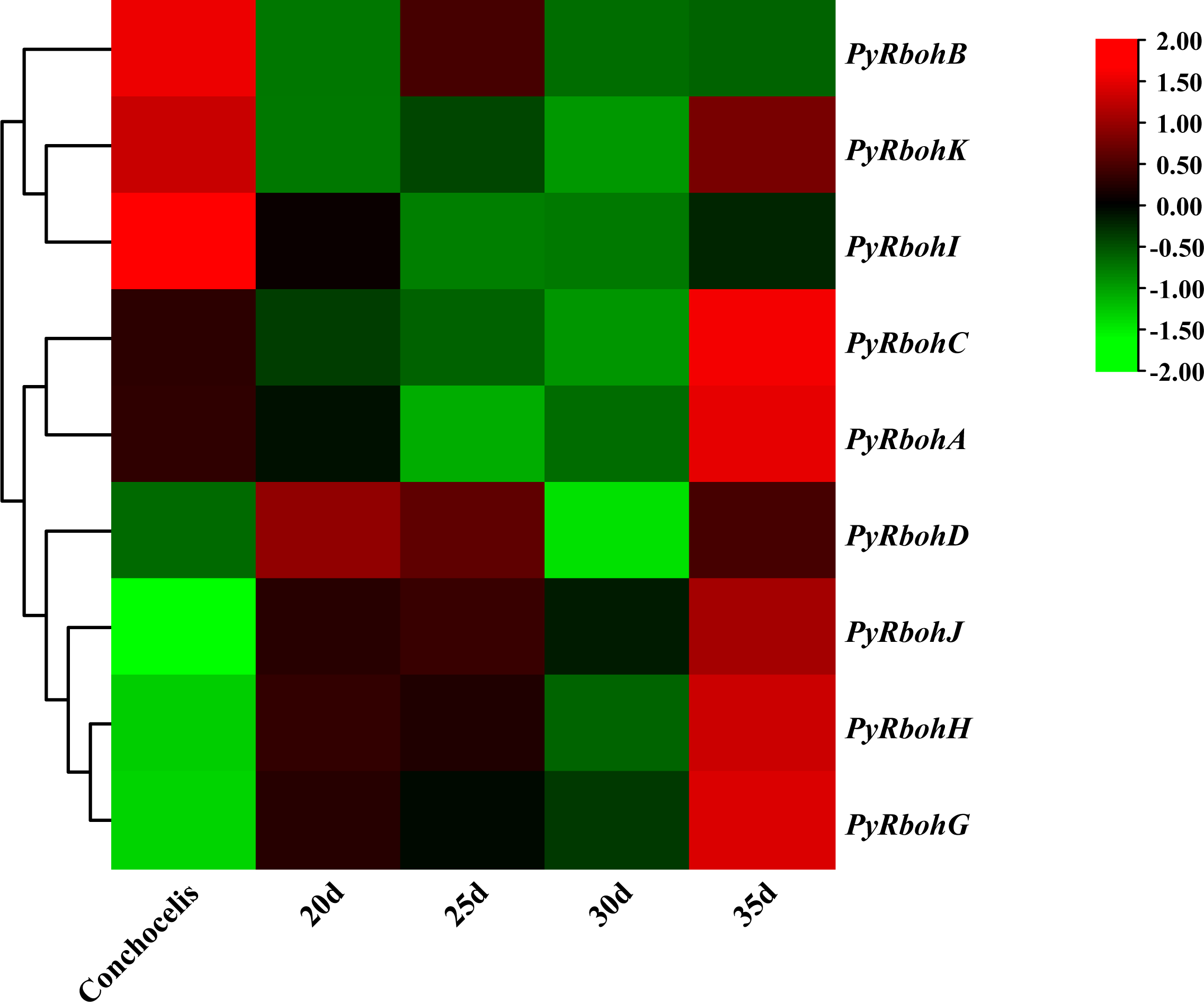

Supplement: Supplementary Figure S1 — Expression profiles of PyRboh genes in conchocelis and different developmental stages of blades in P. yezoensis. [file Image_1.TIF]

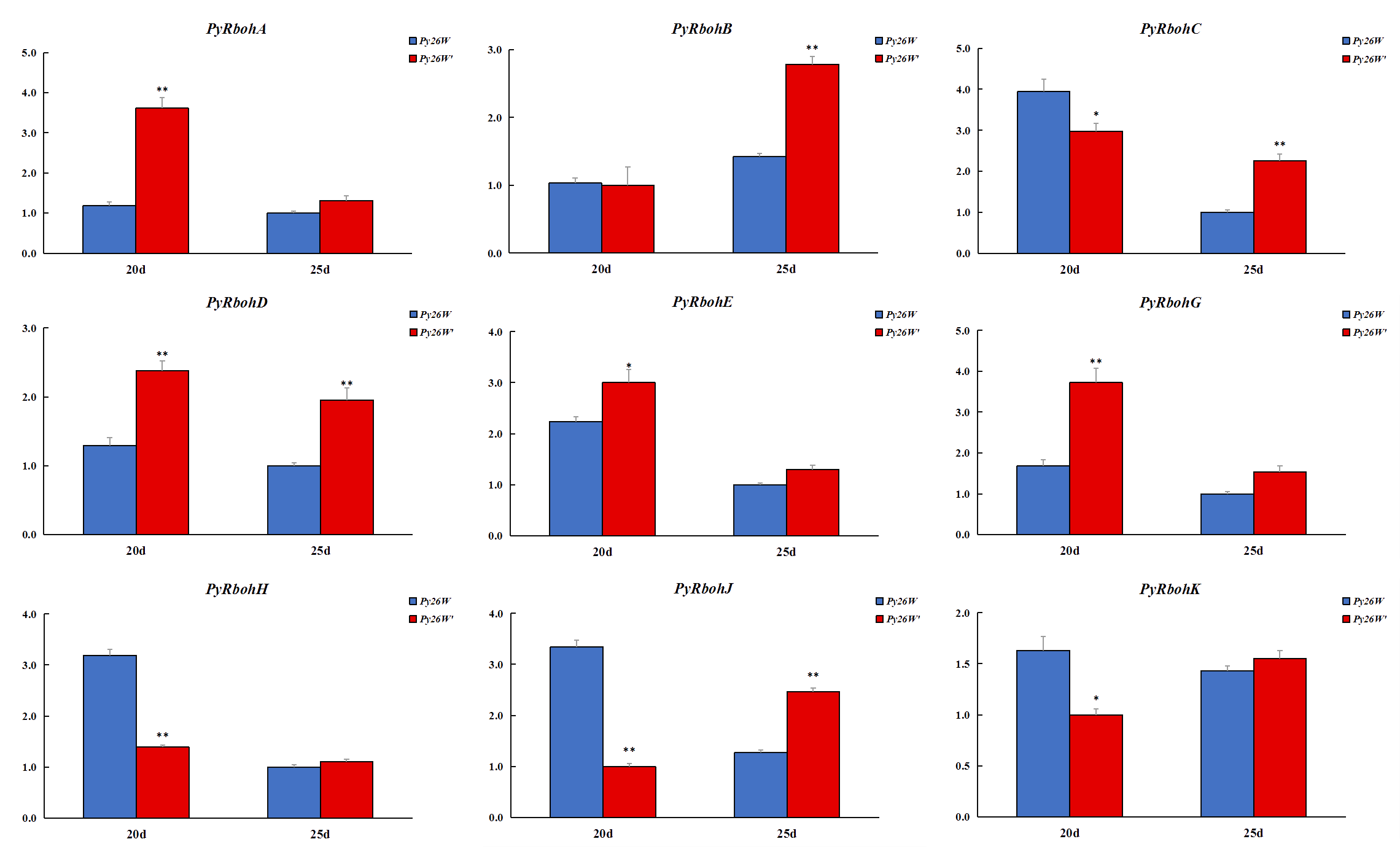

Supplement: Supplementary Figure S2 — Expression profiles of PyRboh genes between Py26W and Py26W' strains at the age of 20 and 25 days, respectively. *Significant difference (P < 0.05); **Highly significant difference (P < 0.01). [file Image_2.TIF]

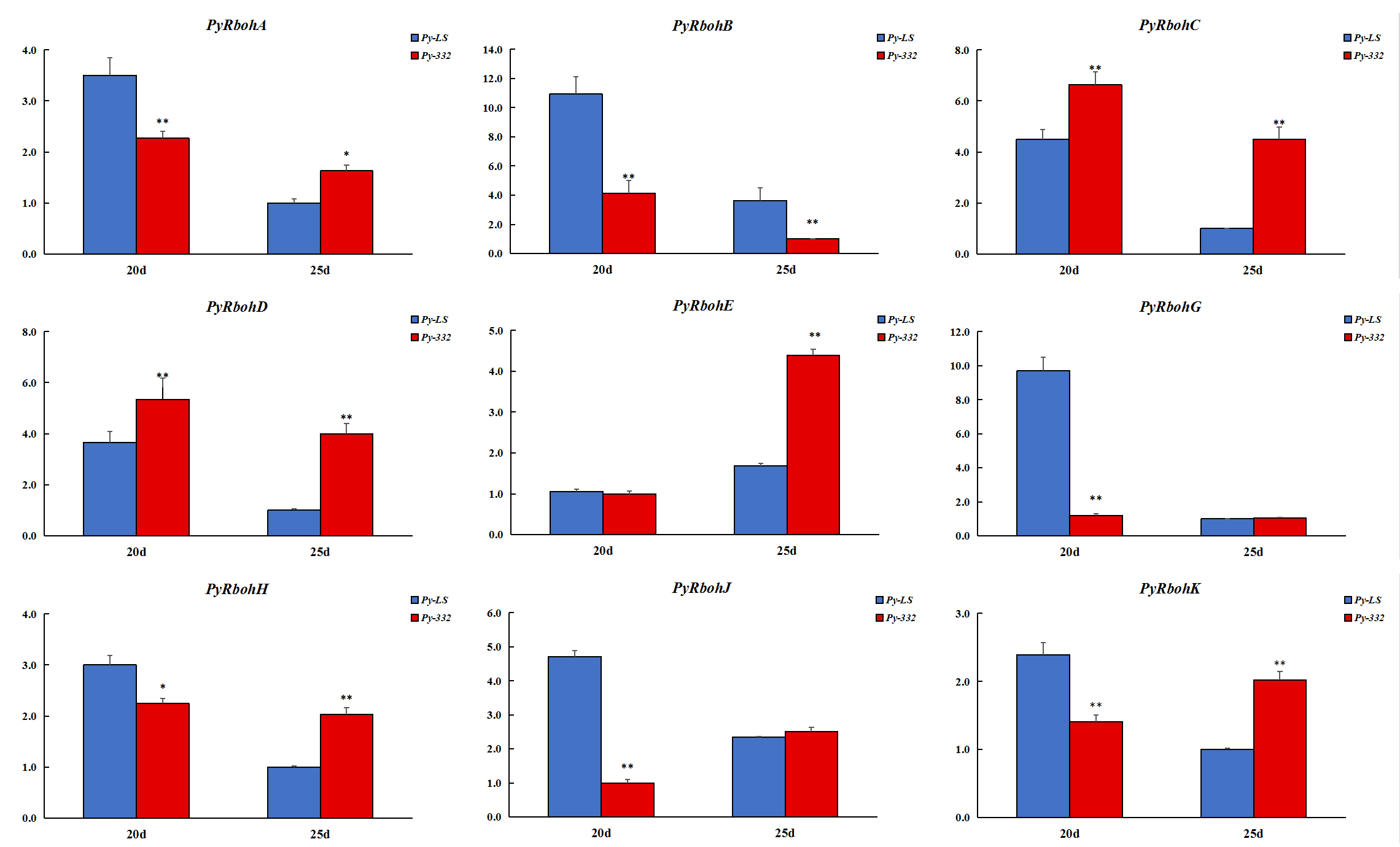

Supplement: Supplementary Figure S3 — Expression profiles of PyRboh genes between Py-LS and Py-332 strains at the age of 20 and 25 days, respectively. *Significant difference (P < 0.05); **Highly significant difference (P < 0.01). [file Image_3.TIF]
